# Elucidating the Interaction of Indole-3-Propionic Acid and Calf Thymus DNA: Multispectroscopic and Computational Modeling Approaches

**DOI:** 10.3390/foods13121878

**Published:** 2024-06-14

**Authors:** Yushi Wei, Dan Zhang, Junhui Pan, Deming Gong, Guowen Zhang

**Affiliations:** State Key Laboratory of Food Science and Resources, Nanchang University, Nanchang 330047, China; weiyushi99@163.com (Y.W.); zd265819036@163.com (D.Z.); panjunhui@ncu.edu.cn (J.P.); dgong01@gmail.com (D.G.)

**Keywords:** computer simulation technology, DNA, groove binding, multispectroscopic methods, plant growth regulator

## Abstract

Indole-3-propionic acid (IPA) is a plant growth regulator with good specificity and long action. IPA may be harmful to human health because of its accumulation in vegetables and fruits. Therefore, in this study, the properties of the interaction between calf thymus DNA (ctDNA) and IPA were systematically explored using multispectroscopic and computational modeling approaches. Analysis of fluorescence spectra showed that IPA binding to ctDNA to spontaneously form a complex was mainly driven by hydrogen bonds and hydrophobic interaction. DNA melting analysis, viscosity analysis, DNA cleavage study, and circular dichroism measurement revealed the groove binding of IPA to ctDNA and showed that the binding did not significantly change ctDNA confirmation. Furthermore, molecular docking found that IPA attached in the A-T rich minor groove region of the DNA. Molecular dynamics simulation showed that DNA and IPA formed a stable complex and IPA caused slight fluctuations for the residues at the binding site. Gel electrophoresis experiments showed that IPA did not significantly disrupt the DNA structure. These findings may provide useful information on the potential toxicological effects and environmental risk assessments of IPA residue in food at the molecular level.

## 1. Introduction

Plant growth regulators (PGRs) are natural plant hormones extracted from organisms or synthetic compounds that have been widely used in agriculture due to their low costs and their effects in regulating plant growth [1]. However, frequent and heavy use of these substances may lead to residues in vegetables, fruits, and water bodies and jeopardize the safety of the entire ecosystem by way of the food chain [2]. PGRs have been reported to cause hepatotoxicity, endocrine disrupting toxicity, neurotoxicity, and reproductive toxicity in animals through foods or functional accumulation [3]. Yilmaz et al. [4] found that indolebutyric acid, as a plant growth regulator, affected tissues’ antioxidant defense system and produced immunotoxic effects in rats.

Indole-3-propionic acid (IPA), as a plant growth regulator, is readily enriched in food and is not easily degraded by indole acetic acid oxidase; thus, its biological activity can last for a long time [5]. Interestingly, previous studies have extensively reported the benefits of IPA. For example, Abildgaard et al. [6] found that IPA improved blood glucose and increased insulin sensitivity. IPA has been reported to inhibit liver lipid synthesis and inflammatory factors [7]. However, it has recently been found that IPA accumulated in food is potentially harmful to human health. IPA significantly enhanced CCl4-induced liver injury and liver fibrosis by activating hematopoietic stem cells and transforming the growth factor-β1/Smads signaling pathway, aggravating liver inflammation and increasing hepatocyte apoptosis [8]. Konopelski et al. [9] reported that IPA increased blood pressure in hypertensive rats through cardiac and vascular mechanisms. These reports suggested that IPA may share the common potential hazard of plant growth regulators, but there is little information on this aspect, which has aroused our interest in conducting a more comprehensive security assessment of IPA.

As an essential genetic material in life activities, deoxyribonucleic acid (DNA) has a clear structure, characteristics, chemical composition, and biological activity; thus, it is critical to maintain its structural integrity. DNA is the target of a large number of small molecules absorbed by the body from the environment and food [10,11]. The binding of small molecules to double-stranded DNA may cause structural damage or change, which may lead to mutations and affect the function of DNA [12]. Therefore, studying the binding characteristics between small molecules and DNA is helpful to reveal the potential toxicity effects of small molecules and evaluate their safety. There are two kinds of interactions between DNA and ligands, covalent and noncovalent interactions, and there are three non-covalent modes: intercalation binding is the insertion of a ligand between base pairs of DNA, groove binding refers to the interaction between the ligand and DNA groove region, and electrostatic binding happens at DNA’s phosphate groups [13]. In the non-covalent binding mode, non-covalent bonds such as hydrogen bonds are the main driving forces to strengthen and stabilize the combination of small molecules and DNA [14]. In recent years, studies have focused on the binding properties of small molecules with DNA and DNA conformational changes [15,16]. However, to our knowledge, the underlying mechanism of the binding between IPA and DNA is unclear.

The study aimed to investigate the interaction mechanism of IPA and ctDNA through multispectral techniques in conjunction with viscosity study, melting measurement, DNA cleavage analysis, and computer simulation. This study may offer a theoretical basis for comprehending the potential toxicological impacts and environmental risk assessment of PGR accumulation in food.

## 2. Materials and Methods

### 2.1. Materials

IPA (>98%, Aladdin Chemistry Co., Ltd., Shanghai, China) was dissolved in ethanol to obtain its stock solution. ctDNA (Sigma-Aldrich Co., St. Louis, MO, USA) was prepared in NaCl solution. The ratio of absorbance at 260/280 nm was recorded to check its purity. The ratio was 1.87 (between 1.8 and 1.9), implying that the ctDNA solution was sufficiently free from protein. According to the molar absorption coefficient (ε_260_ = 6600 L mol^−1^ cm^−1^), the ctDNA’s concentration was determined to be 2.41 × 10^−3^ mol L^−1^. All stock solutions were prepared in Tris-HCl buffer (pH 7.4).

### 2.2. UV–Vis Absorption Spectra

Using the method of Li et al. [15], the UV–vis absorption spectra of ctDNA (4.84 × 10^−6^ mol L^−1^), IPA, and their complex were recorded. The ctDNA solution was mixed with IPA in 1:1 ratio. The UV–vis absorption spectra of IPA after the interaction were obtained by subtracting the spectra of ctDNA alone from its complex (IPA–ctDNA) spectra.

### 2.3. Fluorescence Spectra

A F-7100 fluorescence spectrometer (Hitachi, Tokyo, Japan) was utilized to determine the fluorescence spectra of the ctDNA and the complex at different temperatures (25, 31, and 37 °C). A series of equal-volume (3.2 μL) ctDNA solution (7.23 × 10^−3^ mol L^−1^) was sequentially titrated into the IPA solution (3.28 × 10^−6^ mol L^−1^). The fluorescence emission spectra of the samples were measured at the excitation wavelength of 282 nm, and the slit width was 2.5 nm for both emission and excitation. The measured fluorescence data were corrected as follows [15]:

(1) $$F_{a}=F_{m}\cdot e^{(A_{\mathrm{ex}}+A_{\mathrm{em}})/2}$$

where *F*_m_ and *F*_a_ denote the experimental and after-correction fluorescence data, respectively. *A*_ex_ and *A*_em_ express the absorbance of the ctDNA solution at excitation and emission wavelengths, respectively.

### 2.4. Potassium Iodide Quenching Measurement

The assays of iodide quenching were carried out in IPA and IPA–ctDNA systems by dropping KI (0, 0.33, 0.66, 0.99, 1.32, 1.64, 1.96, 2.28, 2.60, 2.91, and 3.23 × 10^−2^ mol L^−1^). The IPA’s concentration was set at 2.46 × 10^−6^ mol L^−1^, and the concentration of ctDNA was set at 6.28 × 10^−5^ mol L^−1^. The quenching constant (*K*_sv_) was determined to assess the interaction between IPA and ctDNA [17].

### 2.5. Effects of Native or Denatured ctDNA on IPA

The native ctDNA solution was heated at 100 °C for 15 min, then rapidly cooled in an ice water bath for 10 min to obtain the single-stranded ctDNA (ss ctDNA). A solution of ds ctDNA (native) or ss ctDNA (denatured ctDNA) was titrated into the IPA (3.28 × 10^−6^ mol L^−1^). The *K*_sv_ value was calculated based on the fluorescence spectra [18].

### 2.6. Determination of Ionic Strength

The absorbance of ctDNA and ctIPA–DNA at 258 nm was determined by titration with various concentrations of NaCl (0, 0.33, 0.67, 1.00, 1.33, 1.67, 2.00, 2.33, 2.67, 3.00, and 3.33 × 10^−2^ mol L^−1^) [19]. The concentrations of ctDNA and IPA were set at 3.62 × 10^−5^ mol L^−1^ and 3.45 × 10^−5^ mol L^−1^, respectively.

### 2.7. Thermal Melting of ctDNA

The ctDNA and IPA–ctDNA were placed in a water bath with a temperature range of 20–100 °C, and the absorbance values of different systems at 258 nm were recorded at 5 °C intervals. The single-strand fold (*f*_ss_) value was determined by the following formula: *f*_ss_ = (*A* − *A*_20_)/(*A*_100_ − *A*_20_). Here, *A*, *A*_2_, and *A*_100_ are the absorbance values of the corresponding temperatures of 20 °C and 100 °C [20]. The denaturation curve was obtained by plotting *f*_ss_ to T, and the temperature corresponding to *f*_ss_ = 0.5 was the melting point temperature of ctDNA (*T*_m_).

### 2.8. Viscosity Measurements

Viscosity was determined with the Ubbelohde viscometer, and the concentration of ctDNA was set at 2.73 × 10^−5^ mol L^−1^. The flow times of samples through the capillary were measured three times in the absence and presence of IPA/H33258 (0, 0.33, 0.66, 0.98, 1.31, 1.64, 1.96, 2.29, 2.61, 2.94, and 3.26 × 10^−5^ mol L^−1^) to obtain the average flow time (*t*), calibrated with the time for Tris-HCL flow alone (*t*_0_). The value of corresponding viscosity (*η*) was obtained by the formula *η* = (*t* − *t*_0_)/*t*_0_, then plotted with (*η*/*η*_0_)^1/3^ as the vertical coordinate and the concentration ratio ([IPA/Hoechst 33258]/[ctDNA]) as the horizontal coordinate. *η* and *η*_0_ denote the viscosities of IPA–ctDNA or Hoechst 33258-ctDNA complex and ctDNA alone, respectively [21].

### 2.9. Circular Dichroism (CD) Spectra

The CD spectra of the ctDNA solution (2.41 × 10^−4^ mol L^−1^) and IPA–ctDNA mixture with molar ratios of the ctDNA to IPA of 1:10 and 1:5 were measured in the 220–320 nm wavelength range at 25 °C with a MOS 450 circular dichroism spectrometer (Bio-Logic, Claix, France), and three scans were taken in all experiments.

### 2.10. DNA Cleavage

A series of concentrations of IPA (0.98, 1.95, 3.91, 7.81, 15.63, and 31.25 × 10^−4^ mol L^−1^) were incubated with pUC18 plasmid DNA at 37 °C for 2 h. Plasmid DNA bands were stained with Gold View, and 6 μL of loading buffer was mixed thoroughly for each sample, resulting in a final volume of 40 μL for each sample. Then, 10 μL of the samples was added dropwise to 0.8% agarose gel, and the samples were run in 1 × Tris-acetate-EDTA buffer for 30 min. After electrophoresis, the DNA banks were observed under UV light [20].

### 2.11. Molecular Docking

The crystal structure 1BNA of DNA was taken from the Protein Data Bank database, and the 3D structure of IPA was from the PubChem database. Molecular docking simulation was performed using Discovery Studio 3.5 (BIOVIA, San Diego, CA, USA). Water from ctDNA was removed and the necessary hydrogen atoms and charges added before docking. 1BNA and IPA were defined as receptor and ligand, respectively, then, the number of hotspots was set at 100 and the docking tolerance was 0.25. LibDock algorithm was employed to perform the docking calculations. The optimal binding mode of IPA to 1BNA was determined by selecting the highest LibDock score docked conformation [22].

### 2.12. Molecular Dynamics (MD) Simulation

The MD simulation (GROMACS 5.1) technique was utilized to study the dynamic variations of the DNA and IPA–DNA complex structures. The solvent was removed from ctDNA, and the AMBER99SB force field was applied in MD studies; each system was dissolved in a solvent box with a minimum width of 1 nm. The addition of 11 Na^+^ was used to keep the solvation system electrically neutral, and the energy of the system was minimized. In the NVT and NPT ensemble, each system was simulated for 100 ps. Subsequently, the systems were carried out for 100 ns. Finally, periodic boundary conditions were invoked in the simulation [23].

### 2.13. Statistical Analysis

All experiments were made in triplicate, and the results were represented as mean ± standard deviation. One-way analysis of variance (ANOVA) was used to examine the data and the Duncan test was utilized to determine the statistical significance (*p* < 0.05) of the results.

## 3. Results and Discussion

### 3.1. UV–Vis Absorption Spectra

**Figure 1:**
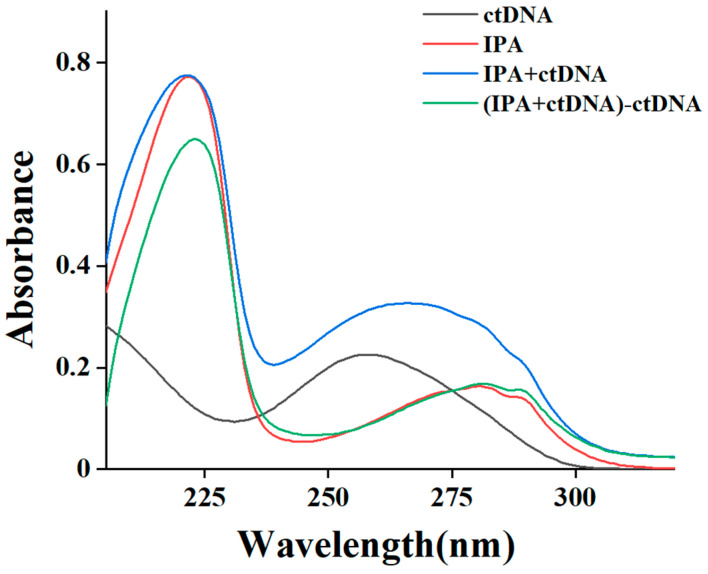
UV spectra of ctDNA (grey line), IPA (red line), IPA–ctDNA complex (blue line), and IPA–ctDNA complex deducting ctDNA (green line).

A preliminary in vitro assessment of the binding pattern of small molecules to ctDNA, intercalation, or groove or external binding can be made by tracking the changes in ctDNA absorbance value and the position of the absorption bands by UV–vis spectra [24]. The interaction between small molecules and ctDNA can change the absorbance or special peak position of small molecules. The intercalation binding mode was reportedly characterized by a significant redshift (more than 10 nm) after the interaction of small molecules with ctDNA. This may be due to the combination of the π * orbital of the ligand with the π orbital of the DNA base pair, which reduced the π-π * transition energy, thus causing a significant absorption redshift. In contrast, the interaction of the groove and external binding modes exhibited a slight spectral shift [25]. The maximum absorption of ctDNA near 258 nm (Figure 1) was associated with chromophores in purine and pyrimidine electron transition. In the absence of ctDNA, IPA has sharp absorption peaks near 220 nm and a shoulder peak near 280 nm, which were considered as its special absorptions [26,27]. The absorption spectra of IPA after the interaction (green line) was obtained by subtracting the spectra of free ctDNA (grey line) from the complex (blue line), and the absorbance near 220 nm was lower than that of IPA before the interaction (red line), accompanied by a 2 nm redshift, suggesting that ctDNA had some effect on the stability of the IPA aromatic ring. A small redshift of the peak position and a slight change in the absorbance value were observed for the absorption peak of IPA near 280 nm, indicating that the IPA–ctDNA complex was formed by groove binding or electrostatic interactions rather than the intercalative mode [28]. Similarly, Li et al. [15] found that the absorbance of 4–octylphenol (OP) at 280 nm decreased after the interaction with ctDNA, but the peak position did not change significantly, which ruled out the possibility of the intercalation mode between OP and ctDNA.

### 3.2. Fluorescence Spectra

**Figure 2:**
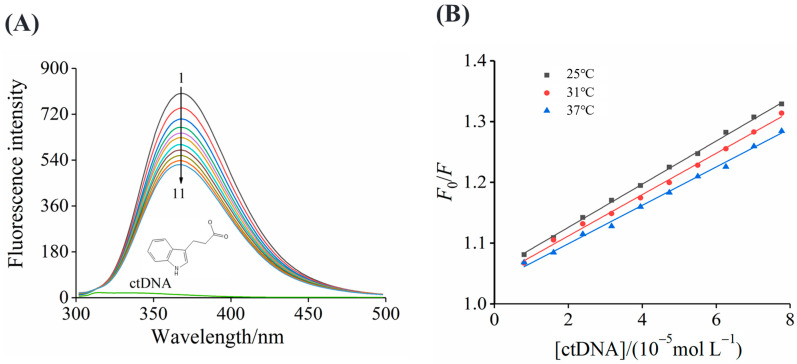
The fluorescence spectra of ctDNA quenching for IPA (**A**) and the Stern–Volmer plots of IPA with ctDNA (**B**).

The intrinsic fluorescence intensity of ctDNA was weak; thus, the fluorescence spectra of IPA at different temperatures were utilized to investigate the binding properties between IPA and ctDNA. The maximum fluorescence peak of IPA was around 367 nm when the excitation wavelength was 282 nm. With the addition of ctDNA, the fluorescence intensity of IPA reduced obviously, but the shape of the maximum peak and the position remained unchanged (Figure 2A), indicating that IPA was bound to ctDNA to form a complex through non-covalent bonding. Geng et al. [28] also found the non-covalent binding of 3-deoxy-3-azaguanosine to ctDNA through detecting the fluorescence intensity of 3-deoxy-3-azaguanosine after the addition of ctDNA.

The mechanism of fluorescence quenching is primarily divided into the dynamic and the static mechanism. Dynamic quenching occurs in the contact between quencher and fluorophore during the transient existence of the excited state, and high temperatures can enhance the degree of collision quenching and diffusion, which is conducive to the formation of a complex by the dynamic quenching mechanism of small molecules and ctDNA [29]. The fluorescence spectra data at different temperatures were described based on the following Stern–Volmer equation:

(2) $$\frac{F_{0}}{F}=1+K_{\mathrm{sv}}[Q]=1+K_{q}\tau_{0}[Q]$$

where *F*_0_ and *F* denote the fluorescence intensities after calibration of IPA and the IPA–ctDNA complex, respectively; τ_0_ denotes the fluorescence life expectancy, which is usually 10^−8^ s [30]; and [*Q*] denotes the concentration of ctDNA.

All of the curves (Figure 2B) showed a good linear relationship, suggesting a single static or dynamic mechanism of ctDNA quenching for IPA. The *K*_sv_ values of the ctDNA–IPA system were decreased from (3.55 ± 0.06) × 10^3^ L mol^−1^ to (3.16 ± 0.08) × 10^3^ L mol^−1^ (Table 1) with increasing temperature from 25 °C to 37 °C. The reduced *K*_sv_ values with the temperature increasing and the *K*_q_ value of ctDNA to IPA was much greater than the maximum scattering collisional quenching constant [2.0 × 10^10^ (L/mol·s)], indicating that the quenching mechanism of IPA to ctDNA had a static manner due to the formation of the ground state complex. This result was similar to the report of Ponkarpagam et al. [31], who reported that ctDNA quenched the fluorescence of rosiglitazone via the static quenching mechanism. In addition, the quenching constant (*K*_sv_) of the interaction between IPA and ctDNA was significantly lower than that of intercalators, such as nile blue 2-tert-butyl-4-methylphenol (1.3 × 10^4^ L mol^−1^), suggesting the relatively lower affinity of IPA than the other intercalators and that IPA may be a groove agent for ctDNA [32].

### 3.3. Binding Constants

The fluorescence results were used to obtain the binding constants (*K*_a_) and the number of binding sites (*n*) using Equation (3):

(3) $$\log\frac{F_{0}-F}{F}=n\log K_{a}-n\log\frac{1}{[Q_{t}]-\frac{\left(F_{0}-F)[P_{t}\right]}{F_{0}}}$$

where [*Q*_t_] and [*P*_t_] denote the concentrations of ctDNA and IPA, respectively. *n* values at different temperatures tended to be 1, indicating that the ratio of IPA to ctDNA in the complex formation was 1:1 [33]. The *K*_a_ values of the IPA–ctDNA interaction decreased with increasing temperature; specifically when the temperature was increased from 25 °C to 37 °C, the *K*_a_ value decreased from 1.75 × 10^4^ to 1.27 × 10^4^ L mol^−1^ (Table 1), implying that at higher temperatures, the stability of the IPA–ctDNA complex decreased. The size of the *K*_a_ value can also be used to determine the binding mode. Notably, in terms of groove binding, the magnitude of *K*_a_ was generally 10^3^–10^4^ M^−1^, and these *K*_a_ values were much lower than the *K*_a_ value of ethidium bromide (a DNA intercalator) reported by Garbett et al. [34] but were the same order of magnitude as that of the groove agent Hoechst 33258 [35]. Meanwhile, Zhang et al. [33] also reported the *K*_a_ value of the minor grooving binder capecitabine with DNA as 1.0 × 10^5^ M^−1^, similar to the results of the present experiments, which supports the inference of IPA as a non-embedding binder. This result suggests that the combination of IPA and ctDNA may be groove binding, but to more accurately determine the binding mode, more experimental data are needed.

### 3.4. Thermodynamics

With the aim of describing the binding forces between IPA and ctDNA at three temperatures, the thermodynamic parameters were obtained from the following equations:

(4) $$\log K_{a}=-\frac{\Delta H{}^{\circ}}{2.303RT}+\frac{\Delta S{}^{\circ}}{2.303R}$$

(5) ΔG°=ΔH°−TΔS°

where R is the universal gas constant. The negative values of Δ*G*° (Table 1) indicated that the reaction process was a spontaneous process. Δ*H*° < 0 and Δ*S*° > 0, obtained by thermodynamic analysis, indicated that the hydrogen bond and hydrophobic interaction were the main driving forces in the reaction between IPA and ctDNA. In addition, the Δ*H*° value (−20.68 kJ mol^−1^) made a great contribution to Δ*G*°, obtained for the IPA–ctDNA interaction, excluding involvement of electrostatic interactions [36]. This result was similar to the major binding forces of procaine to ctDNA reported by Ali et al. [37].

### 3.5. Iodine Ion Quenching

**Figure 3:**
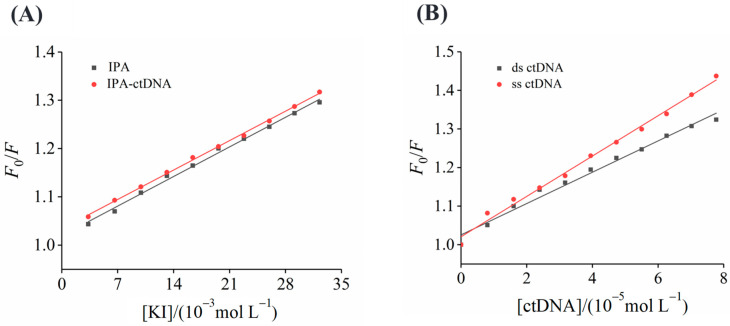
(**A**) Quenching effects of KI on IPA and the IPA–ctDNA complex. (**B**) Stern–Volmer plots of the fluorescence quenching of ds ctDNA and ss ctDNA quenching for IPA.

The iodide ion quenching experiment offers another way to analyze the interaction between IPA and ctDNA. The iodine ion has the ability to quench the fluorescence of small molecules in aqueous medium because it is a highly negatively charged quencher. Insertion agents are well protected from being quenched by iodide ions because they can penetrate inside the DNA helix. However, electrostatically bound molecules and groove binders are easily quenched by iodide ions. The KI’s fluorescence quenching degree on IPA alone was comparable to that of the IPA–ctDNA complex (Figure 3A), and the *K*_sv_ values of IPA in the absence and presence of ctDNA were 8.76 ± 0.21 and 8.70 ± 0.12 L mol^–1^, respectively. The negligible difference in the *K*_sv_ values suggested a groove binding mode between IPA and ctDNA. Cao et al. [38] reported that the chromophore of the intercalating agent formed a sandwich structure with the two base pairs of DNA and the polyphosphate anion skeleton of DNA, thereby preventing the quenching effect of the anion quencher on the small molecules of the intercalating agent, which further illustrated that IPA was a non-embedding agent. The quenching of IPA by iodide ions was hardly affected by the binding of IPA to ctDNA. This may be because when the concentration of iodide ions increased, groove-bound molecules could be released from the groove in a controlled manner, and thus iodide ions could still quench the IPA bound to the ctDNA groove [25]. Shi et al. [39] also found a similar quenching between neotame and ctDNA.

### 3.6. Quenching Effects of ss ctDNA and ds ctDNA

For groove binding agents, unfolded DNA or single-stranded DNA is easier to bind than double-stranded DNA. But for the insertion factor, it is difficult to interact with the loosened DNA. The fluorescence quenching impact of ss ctDNA and ds ctDNA on ligands in the electrostatic binding mode is basically the same. The *K*_sv_ values of ds ctDNA and ss ctDNA were 3.55 × 10^3^ L mol^−1^ and 5.09 × 10^3^ L mol^−1^, respectively; obviously, the ss ctDNA’s *K*_sv_ value was higher (Figure 3B). This result suggested that ss ctDNA had stronger quenching of IPA than ds ctDNA, and IPA bound to the ctDNA’s groove region. Hu et al. [16] investigated the interaction between ss ctDNA and ds ctDNA with benzo[a]pyrene and found that ss ctDNA was more capable of bursting benzo[a]pyrene than ds ctDNA, suggesting the presence of a groove-binding mode and that IPA may act as a grooving agent of ctDNA. This result also supported the result of fluorescence quenching studies.

### 3.7. Ionic Strength

**Figure 4:**
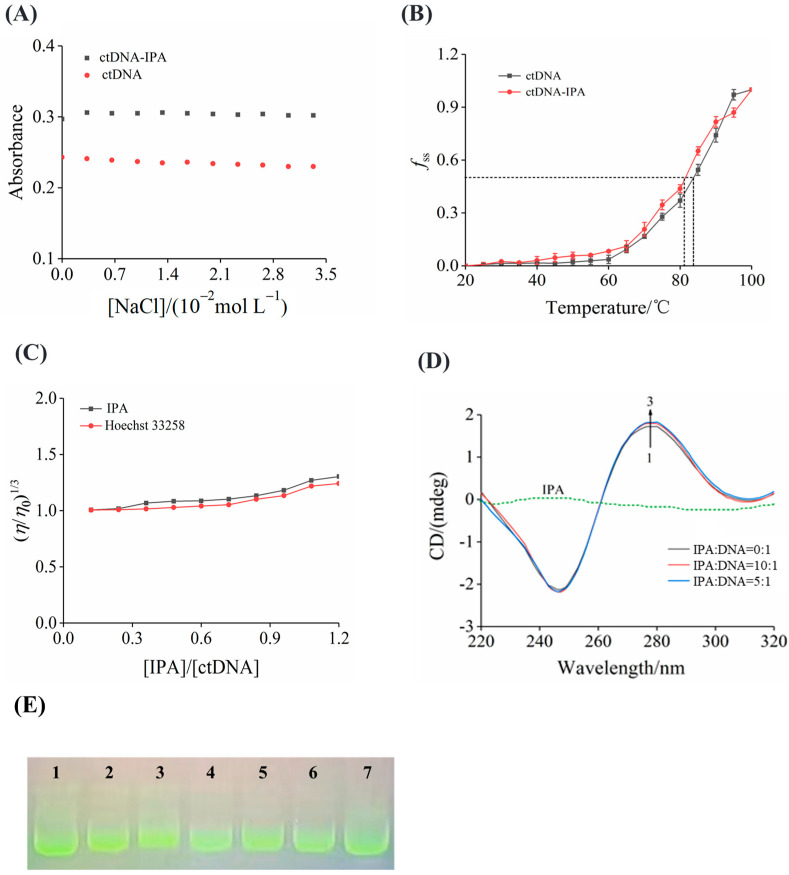
(**A**) Effects of ionic strength on the change in absorbance of free ctDNA and the complex. (**B**) Melting curves of ctDNA in the presence or absence of IPA. *c*(ctDNA) = 4.82 × 10^−5^ mol L^−1^, and *c*(IPA) = 2.46 × 10^−5^ mol L^−1^. (**C**) Effect of IPA or Hoechest 33258 on the relative viscosity of ctDNA. (**D**) CD spectra at different concentration ratios of IPA to ctDNA. (**E**) Gel electrophoresis of DNA strand damage induced by IPA. *c*(IPA) = 0.98, 1.95, 3.91, 7.81, 15.63, and 31.25 × 10^−4^ mol L^−1^.

In the binding of ligands to DNA, electrostatic forces play a subsidiary role because of its weak binding force. If the electrostatic binding interaction dominates the binding process of IPA to ctDNA, the system’s total interaction strength will decrease with the addition of Na^+^ [40]. The effect of ionic strength on the binding process was assessed to verify the possibility of IPA binding to ctDNA by electrostatic binding. The maximum absorption peak of the IPA–ctDNA complex did not significantly change as the concentration of NaCl increased (Figure 4A), indicating that the binding of IPA to ctDNA was unaffected by NaCl. The interaction between IPA and ctDNA did not affect the interaction between positively charged Na^+^ and the negatively charged phosphate backbone. In other words, with the increase of Na^+^ concentration, the interaction between IPA and ctDNA cannot be weakened, and IPA may not be released from the IPA–ctDNA complex, resulting in no change in absorbance [41]. These results suggest that there were no electrostatic forces during the binding process of IPA and ctDNA. Li et al. [15] reported a similar finding in the effect of salt on the 4-octylphenol and DNA binding mode. Consequently, electrostatic binding was negligible in the binding process between IPA and ctDNA.

### 3.8. ctDNA Melting Temperature

Thermal denaturation of the DNA experiment can expose the effect of IPA on ctDNA stability by monitoring the melting temperature at 258 nm. *T*_m_ is the temperature at which heating denaturation results in the loss of half of the DNA double helix structure. Intercalation of small molecules into the helix of DNA makes the structure of DNA more stable, leading to an increase in *T*_m_ of around 5 to 8 °C [42], while the non-intercalation binding mode causes an inconspicuous change in the *T*_m_ value. As shown in Figure 4B, the *T*_m_ values of ctDNA in the absence and presence of IPA are 83.5 °C and 81.1 °C, respectively. The binding of IPA to ctDNA caused a decrease in the *T*_m_ value of only 2.4 °C, suggesting that IPA was a groove agent. *T*_m_ did not change significantly, which may be because the interaction between IPA and the ctDNA base pair did not increase the longitudinal length of the ctDNA chain in the groove binding mode [25]. These results were consistent with the effect of the grooving agent chlorogenic acid on the *T*_m_ of ctDNA [35], supporting that IPA may bind to ctDNA as a grooving agent.

### 3.9. Viscosity

The viscosity of the ctDNA solution correlates with the distance between neighboring base pairs in the DNA helical structure. Classical intercalation leads to a lengthening of DNA because base pairs segregate at the intercalation sites, increasing the relative specific viscosity of such solutions [43]. In contrast, in the mode of electrostatic interactions or groove binding, where the ligand binds to the groove region or the phosphate backbone of the DNA, the viscosity of the DNA solution does not significantly change because the length of the DNA strand remains the same [25]. There was only a small increase in relative viscosity (Figure 4C), which may be because the interaction between IPA and ctDNA led to the bending of the ctDNA chain but did not change the DNA chain length. This result was similar to the result produced by the groove binding agent Hoechst 33258 [44]. The results further revealed that IPA acted as a grooving agent to bind to ctDNA, which was supported by a previous report [45] that clomifene interacted with DNA by groove binding without altering DNA viscosity. These results supported the groove binding of IPA observed in other studies discussed above.

### 3.10. CD Spectra

Free ctDNA can produce a positive band formed by base pair stacking and a negative band caused by right-handed helicity at 275 nm and 245 nm, respectively. These bands are thought to be highly sensitive to the interaction of ligands with ctDNA. After the small molecule was inserted into DNA, the CD spectra showed that the negative and positive peaks of DNA were enhanced due to the elongation and distortion of the DNA helix. This was due to the obvious changes in base stacking and right-handed helicity, and the secondary structure of ctDNA was obviously disturbed, resulting in the change of DNA characteristics in the CD spectra [46]. However, there was insignificant variation in the CD spectra of ctDNA in the case of groove binding or electrostatic interaction [47]. The gradual addition of IPA to ctDNA led to a slight perturbation of the CD spectra (Figure 4D), which further confirmed the groove binding mode of IPA and ctDNA. In addition, aslight rise was noticed in the positive band with increasing IPA concentration. This may be caused by a slight increase in the base stacking degree of ctDNA, which meant that the double helix structure of ctDNA was more stable to a certain extent, but ctDNA still maintained the B-form after binding to IPA. These observations were indicative of IPA bound in preference to the ctDNA’s minor groove region, and the findings were consistent with the interaction of psoralen and ctDNA [48].

### 3.11. DNA Cleavage Activity of IPA

The effect of different concentrations of IPA on converting superhelical pUC18 plasmid DNA into open circular DNA was assessed by gel electrophoresis. Under normal circumstances, intact plasmid DNA (circular) is a relatively faster-migrating superhelical form (Form I); after a single phosphodiester bond has been cleaved, the superhelix should relax to produce a slower-moving incised circular form (Form II); however, if both strands of the superhelical plasmid are cleaved, a linear form (Form III) that migrated slower than Form I but faster than Form II should be generated [40]. The natural pUC18 plasmid DNA only exhibited the Form I band (lane 1), with increasing concentrations of IPA (lanes 2 to 7), and the migration of Form I did not transform to Form II and Form III (Figure 4E), indicating that the damage to DNA by IPA was insignificant. Compared with natural pUC18 plasmid DNA, the mobility of DNA treated by IPA decreased slightly but not significantly, suggesting that IPA formed a complex with ctDNA in a groove binding manner and had no noticeable detrimental effect on the ctDNA’s structure [49]. Haris et al. [50] also found that curcumin caused little structural damage to DNA and there was minor groove binding to ctDNA. Quinoline yellow, as a groove binding agent of ctDNA, did not significantly cleave ctDNA [51].

### 3.12. Molecular Docking

**Figure 5:**
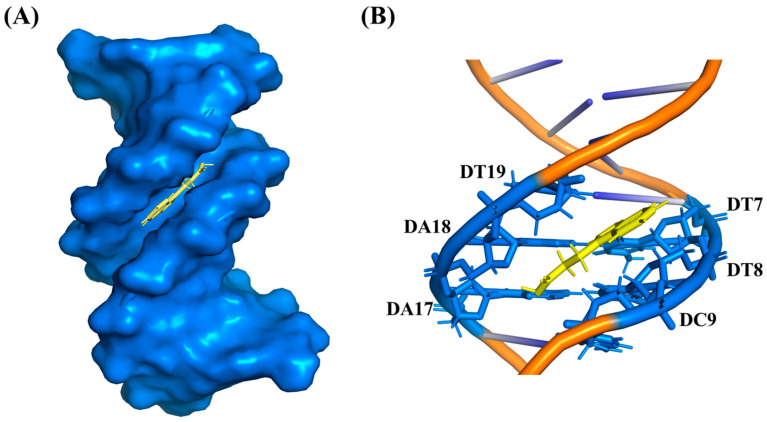
(**A**) The best molecular binding conformation of IPA with 1BNA. (**B**) The docking model with the optimal pose of IPA–1BNA. Blue represents 1BNA and base pairs, yellow represents IPA, and orange in (**B**) represents double strand of 1BNA.

The molecular docking results (Figure 5B) clearly showed that IPA preferentially bound in the AT-rich minor groove region of DNA sequence rather than to the terminal G-C region, and IPA was complemented by the natural curvature of the minor groove of 1BNA to form a mode of groove combination [45] (Figure 5A). Based on our docking studies, hydrogen bonding and hydrophobic interaction played a major role in interaction of IPA with DNA, consistent with the results calculated by thermodynamic parameters. Detailed information on the interaction between IPA and 1BNA is shown in Table 2. Hydrogen bonds were formed between the carboxyl group of IPA and the hydrogen atoms of the A-T base pairs (DA18 on the B-chain, 2.04Å), and its 1-NH group of indole interacted by H-bonding with the 2-carbonyl oxygen of the thymine base in the A-T base pair of duplex DNA (DT7 on the A-chain and DT19 on the B-chain), with bond lengths of 2.18Å and 2.66Å, respectively. In addition, the carbonyl oxygen of IPA formed two weak hydrogen bonds with DT19 (2.71 Å) and DC9 (2.71 Å), and the hydrogen atom on the 2(α) carbon of its indole group formed a weak hydrogen bond with DT8 (2.80 Å); the hydrogen bonding forces between them were weak, probably because the bond lengths were above 2.70Å [52]. In addition, the benzene ring structure in IPA interacted with DT8 to form an additional π-sigma hydrophobic interaction force. Hydrogen bonding interactions and hydrophobic interactions between IPA and 1BNA determined the stability of groove binding. The binding of IPA to 1BNA was similar to that of the groove agent β-amino alcohols [53], binding to the DNA’s A-T rich region, which was probably because the bottom of the G-C sequence was broken by the guanine exocyclic amino group protruding into the groove, preventing the binding of the small groove binder IPA [49]. These molecular docking results further confirmed that IPA was a groove binding agent.

### 3.13. MD Simulation

MD simulation is commonly used to study atomic motion to understand molecular interactions and interpret experimental data. MD calculation is helpful for selecting stable binding structures and identifying the best binding mode. Based on the trajectory analysis, MD simulation was used to further assess the conformational changes of 1BNA in the IPA−1BNA complex.

**Figure 6:**
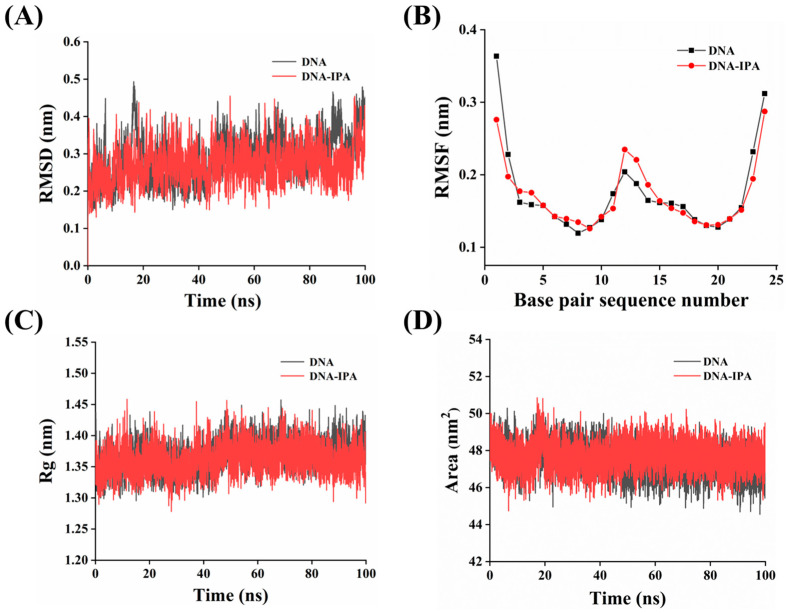
Results of 100 ns MD simulations of IPA with 1BNA: RMSD (**A**); RMSF (**B**); Rg (**C**); and SASA (**D**) plots of free 1BNA and the IPA–1BNA complex.

The root mean square deviation (RMSD) is utilized to assess the stability of simulation systems with regard to simulation time [54]. The intrinsic flexibility and thermal mobility of native 1BNA may be responsible for the observed fluctuations in the RMSD plots of native 1BNA [55]. Initially, the RMSD values of IPA–1BNA gradually increased and fluctuated significantly related to the stability of IPA binding, and then the RMSD values reached equilibrium at 20 ns (Figure 6A), which meant that the IPA–1BNA system reached a steady state. Although the oscillation pattern of the IPA–1BNA complex was very close to that of free 1BNA, the complexes’ average fluctuation intensity (0.270 nm ± 0.05) was less than that of free 1BNA (0.291 nm ± 0.06), increasing the stability of 1BNA [56]. This was consistent with the experimental results of CD spectra.

To further characterize the effects of the dynamic behavior and the structural stability of IPA on 1BNA, the root mean square fluctuation (RMSF) value of free 1BNA and the IPA–1BNA complex were calculated. The average RMSF value of free 1BNA was less than 0.2 nm (0.174 nm ± 0.06) (Figure 6B), indicating that it was structurally stable [57]. The RMSF values of the IPA–1BNA complex were slightly different from natural 1BNA. Specifically, the RMSF values of base pairs 2 to 7 and 12 to 14 (base pair sequence numbers) were increased in the IPA–1BNA complex compared to the native 1BNA fragments, suggesting that base pair fluctuations were increased by binding to IPA. In contrast, in the IPA–1BNA complex, the fluctuations of base pairs 1 to 2, 16 to 19, and 22 to 24 were significantly lower due to the binding of IPA, implying that the interaction between DNA and IPA resulted in a decrease in the flexibility of the residues. In addition, the average RMSF value of the IPA–1BNA complex was 0.171 nm ± 0.04, lower than that of native 1BNA, suggesting that the complex was less volatile. These results emphasize the stability of IPA binding to 1BNA [58].

The radius of gyration (Rg), a commonly used index to evaluate structural compactness, represents the distance from the center to the end of all atoms within a certain period of time [59]. The lower the fluctuation and values of Rg, the higher the densification and stability of the systems. The average Rg value of IPA–1BNA was 1.361 nm ± 0.02 (Figure 6C), smaller than the average Rg value of 1BNA (1.368 nm ± 0.02), suggesting that the helical structure’s compactness was slightly increased after binding IPA to DNA. These results were consistent with the CD spectra results, which suggested minimal conformational changes in 1BNA when IPA bound to the groove of 1BNA [56].

Solvent-accessible surface area (SASA) is defined as the surface area of DNA that is accessible to a solvent probe. The stability of 1BNA was further investigated by analyzing the changes in the values of SASA (Figure 6D). There was a small change in the SASA values of the IPA–1BNA complex. The calculated average SASA value of the IPA–1BNA complex was 47.507 nm^2^ ± 0.74, slightly lower than the average SASA value (47.704 nm^2^ ± 0.79) of native 1BNA. These results provide more evidence for the stability of the IPA–1BNA complex and the groove binding of IPA to 1BNA during the simulation period [55], which is consistent with the other experimental results.

## 4. Conclusions

In this study, the binding mode between IPA and ctDNA was investigated using various spectroscopic methods, viscosity measurement, melting measurement, DNA cleavage study, molecular docking, and MD simulation. IPA was found to be preferentially bound in the AT-rich minor groove region of DNA, resulting in a static quenching of IPA fluorescence. The binding of ctDNA and IPA was primarily mediated by hydrophobic forces and hydrogen bonds, which was supported by molecular docking results. The electrostatic and intercalative mode of binding were ruled out, as confirmed by viscosity measurement, thermal melting measurement and ionic strength assays, iodine ion quenching study, and CD analysis. Furthermore, the molecular docking and MD simulation demonstrated that 1BNA was stabilized by hydrogen bonding and hydrophobic interactions with IPA, and IPA affected the degree of freedom of the 1BNA structure and altered the flexibility of 1BNA base pairs but did not notably change the 1BNA structure. These results are conducive to understanding the mechanism of IPA toxicity in fruits and grains at the molecular level.

## Figures and Tables

**Table 1:** Quenching constants (*K*_sv_), binding constants (*K*_a_), and thermodynamic parameters of IPA interaction with ctDNA.

*T* (℃)	*K*_sv_ (×10^3^ L mol^−1^)	*R* ^a^	*K*_q_ (×10^11^ L mol^−1^ s^−1^)	*K*_a_ (×10^4^ L mol^−1^)	*R* ^b^	Δ*H*° (kJ mol^−1^)	Δ*G*° (kJ mol^−1^)	Δ*S*° (J mol^−1^ K^−1^)
25	3.55 ± 0.06	0.9977	3.55 ± 0.06	1.75 ± 0.10	0.9948	−20.68	−24.27	12.05
31	3.39 ± 0.07	0.9961	3.39 ± 0.07	1.62 ± 0.14	0.9958		−24.34	
37	3.16 ± 0.08	0.9939	3.16 ± 0.08	1.27 ± 0.08	0.9951		−24.42	

*R*_a_ is the correlation coefficient for the *K*_sv_ values; *R*_b_ is the correlation coefficient for the *K*_a_ value.

**Table 2:** The interaction forces of IPA and 1BNA.

Receptor	Ligand	Hydrogen Bonding	Weak Hydrogen Bond	Hydrophobic Interactions
1BNA	IPA	DT7 (2.18Å), DA18 (2.04Å), DT19 (2.66Å)	DT8 (2.80 Å), DC9 (2.71 Å), DT19 (2.71 Å)	DT8 (π-sigma, 2.49 Å)

## Data Availability

The original contributions presented in the study are included in the article, further inquiries can be directed to the corresponding author.
